# Surgical management of complex non-union in chest wall instability: focus on posterior rib fractures, parasternal cartilage rupture, and costal margin injury

**DOI:** 10.1093/jscr/rjag208

**Published:** 2026-03-29

**Authors:** Christopher Spering, Philipp Echterbeck, Corinna Carla Dobroniak, Wolfgang Lehmann, Hassan Awan Malik

**Affiliations:** Department of Trauma Surgery, Orthopedics and Plastic Surgery, University Medical Center Goettingen, Robert-Koch-Str. 40, D-37075 Goettingen, Germany; Department of Trauma Surgery, Orthopedics and Plastic Surgery, University Medical Center Goettingen, Robert-Koch-Str. 40, D-37075 Goettingen, Germany; Department of Trauma Surgery, Orthopedics and Plastic Surgery, University Medical Center Goettingen, Robert-Koch-Str. 40, D-37075 Goettingen, Germany; Department of Trauma Surgery, Orthopedics and Plastic Surgery, University Medical Center Goettingen, Robert-Koch-Str. 40, D-37075 Goettingen, Germany; Department of Trauma Surgery, Orthopedics and Plastic Surgery, University Medical Center Goettingen, Robert-Koch-Str. 40, D-37075 Goettingen, Germany

**Keywords:** rib fracture, non-union, posterior ribs, parasternal cartilage, costal margin rupture, surgical stabilization, thoracic trauma, chest wall instability

## Abstract

Non-union of the chest wall is an underrecognized but functionally significant complication of thoracic trauma, particularly when involving posterior ribs, parasternal cartilage, and the costal margin. Complex instability patterns can result in persistent pain, mechanical dysfunction, and impaired respiration. We describe two patients with symptomatic chest wall non-union. Case 1 was a 62-year-old man with posterior non-union of the seventh and eighth ribs and a secondary costal margin rupture with intercostal hernia. He underwent combined posterior rib plating and costal margin reconstruction, nerve-sparing fibre-tape sutures, and double-layer mesh. Case 2 was a 25-year-old man with a radiographically occult rupture and pseudarthrosis of the third parasternal costal cartilage. He was treated with trans-costosternal osteosynthesis and local bone grafting. These cases illustrate that dynamic and multimodal imaging are often required to diagnose non-unions, and that contemporary plating and mesh techniques can restore stability, relieve pain, and permit early mobilization.

## Introduction

While the majority of rib, costal cartilage, and sternal fractures heal uneventfully, non-union (pseudarthrosis) develops in 5%–10% of cases and can cause disabling chest wall instability [1, 2]. Complex non-unions involving the posterior ribs, parasternal cartilage, and costal margin represent unique diagnostic and therapeutic challenges [1, 3–5]. Posterior rib fractures are particularly prone to missed diagnosis due to overlapping musculature and subtle clinical findings [1, 6, 7], whereas non-union of the parasternal cartilage and costal margin ruptures may present with paradoxical breathing, chronic pain, or focal chest wall deformity but minimal radiographic changes [3, 4, 8].

Non-union is usually defined by absent fracture healing after 3–6 months in combination with persistent clinical and radiological evidence of instability [1, 2]. High-energy trauma, repetitive microtrauma (for example, from severe coughing), and patient-related factors such as osteoporosis, smoking, and poor nutrition increase the risk of pseudarthrosis [1, 3, 9]. In many patients, symptoms include persistent focal pain, mechanical ‘clicking’, or a sense of instability that limits daily activities and work capacity [1–3, 8, 10]. Surgical stabilization is indicated in patients with non-union causing persistent disabling pain, mechanical instability, functional limitation, herniation/visceral injury, or failure of previous repair [1, 2, 7–9, 11–15].

Despite increasing experience with surgical stabilization of rib fractures, posterior rib pseudarthrosis, parasternal cartilage rupture, and costal margin non-union remain underreported entities with distinct diagnostic and technical considerations [1, 3–5]. We present two illustrative cases to highlight the role of multimodal imaging, including dynamic ultrasound, and contemporary osteosynthesis techniques in their management [4, 5, 13].

## Case reports

### Case 1

Posterior rib non-union of the seventh and eighth rib with subsequent costal margin rupture

#### Patient and initial presentation

**Figure 1:**
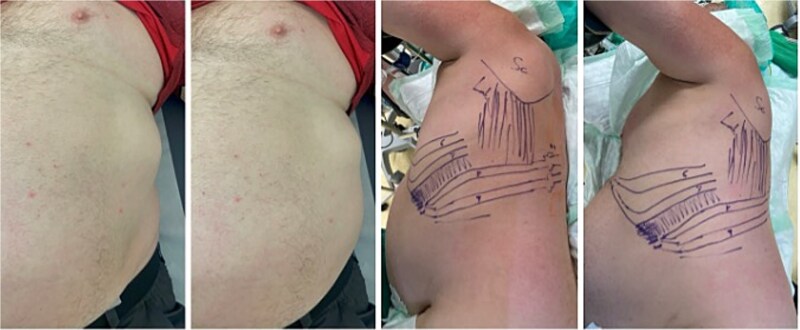
Preoperative images of the patient in the chest wall outpatient clinic (left) presenting with persistent deep thoracic back pain, mechanical ‘clicking’, and an intermittent bulge at the left costal margin during coughing. The two right images show the mapping of the injury pattern after indication for surgery, positioning in lateral right position and intra-op ultrasound detecting of the non-unions of the posterior column of the eighth and ninth rib as well as the costal margin rupture anterolateral between the seventh and eighth rib.

A 62-year-old man sustained multiple left-sided rib fractures (ribs 6–9) in a high-energy bicycle accident and was initially treated conservatively. Six months later, he presented to our chest wall outpatient clinic with persistent deep thoracic back pain, mechanical ‘clicking’, and an intermittent bulge at the left costal margin during coughing (Fig. 1).

On examination, there was focal tenderness over the posterior axillary line and paradoxical movement at the left costal margin during deep inspiration and coughing. The patient reported marked limitations in physical activity and work capacity due to pain and instability.

#### Diagnostic work-up

**Figure 2:**
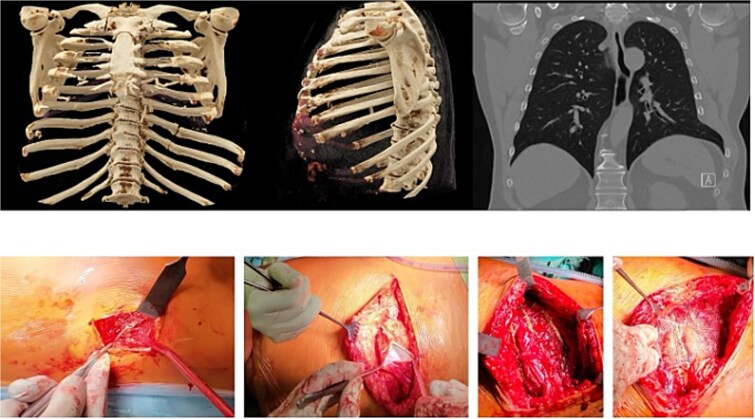
3D-CT reconstruction of the thorax with fractured ribs 8 and 9 on the left posterior column of the chest wall. Also shown in the coronal view of the pre-operative CT scan is shown the costal margin rupture (right upper image) between the seventh and eighth rib anterolateral with a herniation of the lung and the diaphragm. The bottom images show intraoperative images of the preparation of the surgical field: left: debridement of the eighth and ninth rib non-union and bone grafting the gap. Second left: dissection of the lungs herniation, diaphragm, and peritoneum in the anterolateral costal margin rupture. Third left: fixing and reconstruction of the costal margin with using KLS Martin flexible plates with 7 mm angulated screws, as well as applying plates to the unfractured sixth and seventh rib as well as eighth and ninth rib, to prevent the fibre tapes from cutting in the ribs when reconstruction of the intercostal rupture. Right image: applying an additional mesh to securely fix the ribs towards each other and prevent secondary redislocation as well as spreading the force.

Thin-slice computed tomography (CT) of the chest with multiplanar and three-dimensional reconstruction demonstrated non-union (pseudarthrosis) of the left seventh and eighth posterior ribs with sclerosis and displacement, as well as a defect at the costal margin between the seventh and eighth ribs with herniation of lung and diaphragm into the intercostal space (Fig. 2). This pattern was consistent with a costal margin rupture associated with intercostal hernia, according to the Sheffield classification of costal margin injuries.

Dynamic ultrasound was performed with the patient in the lateral decubitus position. Sonography confirmed macromovement at the costal margin during respiration and coughing, and visualized the herniation of pleural and diaphragmatic tissue between the fractured ribs (Fig. 1).

Routine laboratory investigations were unremarkable. Pulmonary function tests showed reduced forced vital capacity and painful restriction of maximal inspiratory and expiratory manoeuvres.

#### Diagnosis

Based on the clinical and imaging findings, the diagnosis was complex chest wall instability due to posterior rib pseudarthrosis of the seventh and eighth ribs and secondary costal margin rupture with intercostal hernia.

#### Surgical management

After multidisciplinary discussion involving trauma surgery, thoracic surgery, and anaesthesiology, a combined procedure was planned to address both the posterior rib non-unions and the costal margin rupture in a single session.

The patient was positioned in a right lateral decubitus position. Through a muscle-sparing posterior parascapular approach, the seventh and eighth rib non-union sites were exposed. Fibrous tissue and sclerotic bone ends were debrided until healthy bleeding bone was encountered, and the fracture gaps were prepared for bone grafting. Temporary stabilization was achieved with Kirschner wires.

A second, muscle-sparing lateral thoracotomy was then performed over the anterolateral costal margin. The hernia sac was identified, and herniated lung, diaphragm, and peritoneum were carefully reduced into the thoracic and abdominal cavities. The costal arch was reconstructed using buttress plates fixed along the costal margin. Additional flexible plates were applied to the sixth, seventh, eighth, and ninth ribs to prevent fibre-tape sutures from cutting through the ribs during reconstruction of the intercostal defect.

Intercostal nerve-sparing fibre-tape sutures were passed through free plate holes and around the ribs to repair the intercostal rupture while preserving the neurovascular bundles. Finally, a double-layer mesh was attached to the plates to provide additional reinforcement of the reconstructed costal margin and to distribute forces across the repair (Fig. 2). Bone graft harvested from local rib segments was placed into the posterior rib defects before definitive fixation with rigid plates.

Chest drains were inserted, and the wounds were closed in layers. The patient was extubated in the operating room and transferred to the intensive care unit for monitoring.

#### Outcome

The patient reported immediate reduction of pain and disappearance of the mechanical ‘clicking’ and bulging at the costal margin. Early postoperative mobilization and respiratory physiotherapy were initiated on the first postoperative day.

**Figure 3:**
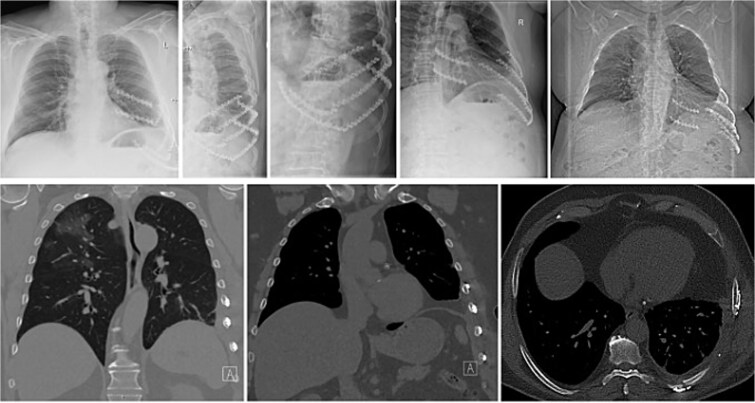
Postoperative X-ray and CT images. The X-ray (top row) shows the two rigid plates (green, solid, KLS Martin) bridging the non-union and bone grafting of the eighth and ninth rib in the posterior column. Additionally the costal margin along the rupture line is fixed with two plates in the cartilage tissue. Four plates (sixth–ninth rib) are applied to the anterolateral column of the sixth to ninth rib to prevent the fibre tape from cutting through the ribs. The bottom images show coronal and axial CT images with the reconstruction of the costal margin as well as the diaphragm and the non-union of the eighth and ninth rib posterior.

At 6-month follow-up, plain radiographs and CT showed bony union of the posterior seventh and eighth ribs, a stable reconstruction of the costal margin, and no recurrence of the intercostal hernia (Fig. 3). Pulmonary function had returned to his pre-injury level, and he had resumed full activities without restrictions. No implant-related complications occurred during follow-up.

### Case 2

Parasternal cartilage rupture of the third rib with pseudarthrosis and indication for trans-costosternal osteosynthesis

#### Patient and initial presentation

A 25-year-old man sustained blunt trauma from a fall. Initial evaluation at an outside hospital included chest radiography, which did not show any rib or sternal fracture, and he was discharged with analgesics.

**Figure 4:**
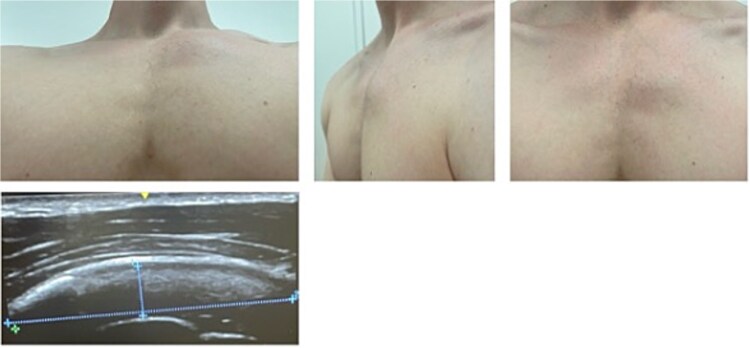
The young patient presenting in the chest wall outpatient clinic with severe pain and swelling of the third parasternal cartilage rib proportion. The ultrasound (bottom image) shows intact muscle tissue of the major pectoralis muscle but swelling just on top of the cartilage tissue.

Three months later, he presented to our chest wall outpatient clinic with persistent, localized anterior chest pain and swelling near the left third costochondral junction. Pain was exacerbated by deep inspiration, coughing, and upper body movements, and had led to prolonged incapacity for work (Fig. 4).

On physical examination, there was a palpable ‘step-off’ and focal tenderness over the left third parasternal rib segment, with subtle chest wall instability on deep inspiration. No clinical evidence of flail chest or gross deformity was present.

#### Diagnostic work-up

An initial thin-slice CT scan of the chest showed an undisplaced lateral fracture of the left ninth rib in an advanced stage of healing and subtle changes around the cartilage of the left third rib near the sternum, but no definite osseous fracture. Magnetic resonance imaging (MRI) of the chest demonstrated fluid and oedema in the soft tissue and periosteum around the left third parasternal cartilage segment without a clear fracture line.

**Figure 5:**
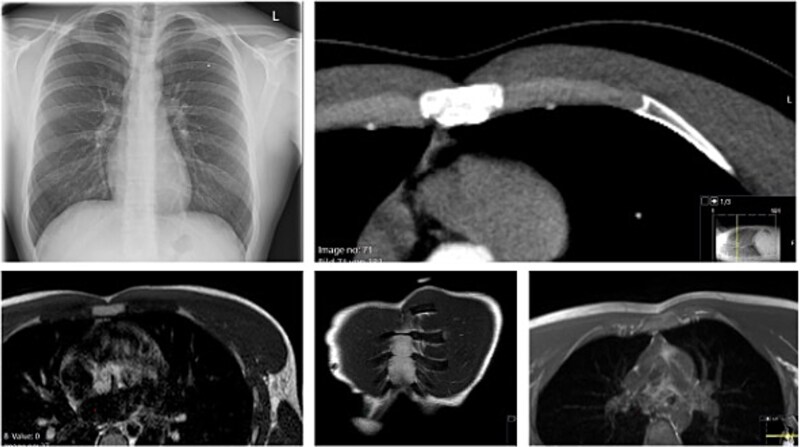
Preoperative imaging with X-ray (left, top) not showing any pathological finding. CT scan reveals a parasternal non displaced rupture of the cartilage. The MRI (bottom three images) proof the rupture of the cartilage and the swelling of the periosteum.

Because of persistent pain and functional impairment, a repeat MRI focused on the left third parasternal region was performed 8 weeks later. This scan suggested a rupture and non-union of the costal cartilage of the third rib at the parasternal junction with displacement and fibrous tissue interposition (Fig. 5).

Dynamic ultrasound revealed swelling over the third parasternal cartilage with preservation of the overlying pectoralis major muscle. During respiration and gentle manual compression, paradoxical micromovement was seen at the cartilage site, consistent with instability, while the peri-cartilaginous fluid persisted (Figs 4 and 5).

Routine laboratory tests did not show any signs of systemic inflammation or metabolic bone disease.

#### Diagnosis

The working diagnosis was parasternal costal cartilage rupture of the left third rib with chronic pseudarthrosis at the costosternal junction.

#### Surgical management

Given persistent disabling pain, local instability, and failure of conservative management over several months, surgical stabilization was recommended. Particular attention was paid to preoperative planning, including selection of MRI-compatible implants to allow future imaging if needed.

**Figure 6:**
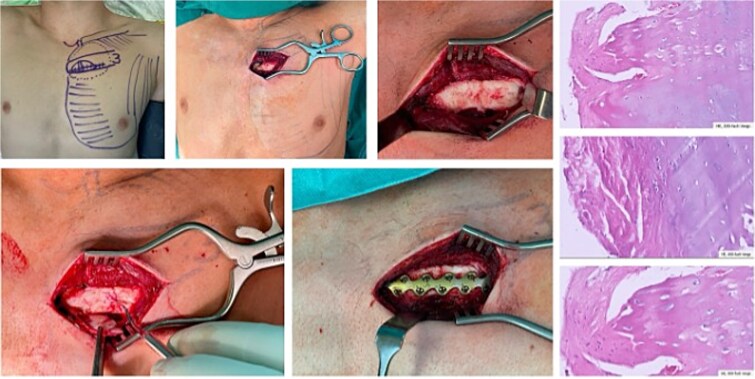
Intraoperative mapping prior to skin incision by using ultrasound. Preparation of the cartilage tissue and proofing the rupture and instability. Debridement of the ‘cartilage non-union’. Bone grafting and applying a bridging plate. The histopathological HE-staining reveals the ‘non-union’of the cartilage rupture by showing regular cartilage tissue and cells as well as fibrinocytes.

Under general anaesthesia, the patient was positioned supine. Using intraoperative ultrasound, the exact location of the cartilage rupture was mapped and marked on the skin (Fig. 6). A small, muscle-sparing incision was made over the left third parasternal region, and the pectoralis major muscle was gently split along its fibres to expose the costal cartilage and sternum.

Fibrous tissue and non-viable cartilage were excised, and the cartilage–bone interfaces on the third rib and sternum were freshened until healthy tissue was encountered. A low-profile locking plate (L1, KLS Martin®) compatible with MRI was contoured to bridge the third rib, costal cartilage, and sternum. The plate was fixed with unicortical screws into the sternum, the costal cartilage, and the osseous portion of the third rib.

Local cancellous bone graft obtained from the adjacent rib was packed around the bone–cartilage junctions to enhance stability and promote healing (Fig. 6). Haemostasis was secured, and the wound was closed in layers without drainage.

Histopathological examination of the resected tissue confirmed chronic cartilage non-union with fibrous tissue and viable cartilage cells, without evidence of malignancy or infection.

#### Outcome

Postoperatively, the patient reported immediate relief of mechanical instability and substantial reduction in pain. He was discharged on the second postoperative day and started gentle range-of-motion and respiratory exercises.

**Figure 7:**
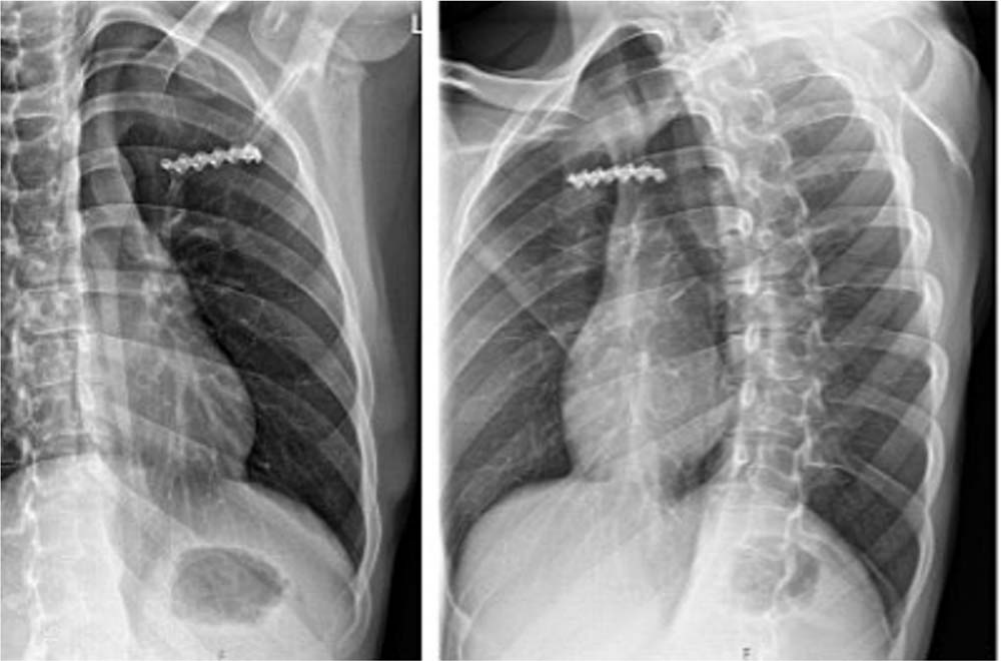
Postoperative X-ray showing the bridging position of the plate on the third rib.

At 3-month follow-up, chest radiography demonstrated a stable bridging plate on the left third rib and sternum with no evidence of hardware loosening (Fig. 7). Clinically, there was no residual swelling or instability, and the patient had returned to full work duties and recreational activities without restriction. No complications or implant-related problems occurred during follow-up.

## Discussion

In both patients, chest wall non-union resulted in persistent pain and mechanical instability despite an initial conservative approach. Case 1 illustrates how posterior rib pseudarthrosis can destabilize the entire hemithorax and precipitate secondary costal margin rupture with intercostal herniation. Case 2 demonstrates that parasternal costal cartilage pseudarthrosis may remain radiographically occult on standard imaging and yet cause significant symptoms and work incapacity in a young patient.

These cases highlight several important diagnostic considerations. Thin-slice CT with multiplanar and three-dimensional reconstructions remains the gold standard for identifying osseous rib non-union, displacement, and deformity, especially in the posterior and costal margin regions [1, 3–5]. MRI is useful for assessing cartilage and soft-tissue pathology, such as costal cartilage fractures, periosteal oedema, and pseudarthrosis in the parasternal region [3]. Dynamic ultrasound offers a complementary, radiation-free modality that allows direct visualization of micromotion at the fracture or non-union site during respiration and manual compression, and is particularly valuable for costal cartilage and costochondral injuries that are not well depicted on radiography or CT [3, 10, 16]. Our findings are consistent with previous reports that sonography is clearly superior to conventional radiography for the detection and follow-up of costal cartilage fractures and non-unions.

From a therapeutic perspective, our experience suggests that individualized operative reconstruction can restore chest wall stability and relieve chronic pain in selected patients with complex non-union. In the first patient, combined posterior rib plating, costal margin buttressing, nerve-sparing fibre-tape repair of the intercostal defect, and double-layer mesh reinforcement provided a durable solution for a Sheffield CMR-IH–type lesion associated with posterior pseudarthrosis [5, 13]. In the second patient, trans-costosternal plating with a low-profile, MRI-compatible locking plate and local bone grafting achieved stable union of a third-rib parasternal cartilage pseudarthrosis and allowed an early return to full function.

### Epidemiology and risk factors

Symptomatic non-union of rib fractures is increasingly recognized as a distinct clinical problem. Although exact incidence data are limited, non-union has been estimated to occur in 5%–10% of rib and sternal fractures in general series, and in about 1.6% of all rib-fracture patients in one institutional cohort [1, 15]. A recent scoping review of 229 surgically treated rib non-unions identified chronic chest pain (93%), mechanical ‘clicking’ (35%), dyspnoea (11%), and chest wall deformity (10%) as the predominant presenting symptoms [17]. Non-union is typically defined as persistent radiographic and clinical instability after at least 3–6 months, consistent with definitions used for long-bone fractures [1, 2, 15].

Risk factors for rib and costal cartilage non-union include high-energy trauma, severe chest wall deformity, and patient-related factors such as smoking, osteoporosis, malnutrition, diabetes, chronic steroid or non-steroidal anti-inflammatory drugs (NSAID) use, and vitamin-D deficiency [1, 3, 7, 17]. Repetitive microtrauma from chronic cough is an emerging aetiology, particularly in older or osteoporotic patients, and has been associated with both rib non-union and costal margin rupture [6, 18, 19]. In costal cartilage injuries, healing appears to be inherently less predictable than in bone: a prospective follow-up of blunt-trauma costal cartilage fractures showed that most lesions calcified and became stable over time, but a minority developed persistent instability or non-union with ongoing symptoms [3].

### Indications and timing for surgical stabilization

Most patients with rib and costal cartilage fractures recover with optimized conservative therapy, including multimodal analgesia, respiratory physiotherapy, and—when indicated—regional anaesthesia [20–22]. Surgery is reserved for a symptomatic subset. Across case series and the recent Chest Wall Injury Society (CWIS) recommendation for non-united rib fractures, the main indications for operative stabilization are:

- Persistent, disabling pain and/or mechanical instability (clicking, paradoxical motion) despite at least 3–6 months of conservative management;
- Objective non-union of one or more ribs or costal cartilage segments on imaging, with corresponding clinical tenderness and instability;
- Associated respiratory impairment, chest wall deformity, or flail segment;
- Costal margin rupture with intercostal hernia, risk of visceral injury, or failure of previous repair;
- Symptomatic sternal or parasternal non-union after trauma or sternotomy [1, 2, 11, 12, 20, 23].

The CWIS guideline for surgical stabilization of non-united rib fractures (SSNURF) concluded, based on low-quality but consistent evidence, that surgery can decrease pain, reduce opiate use, and improve patient-reported outcomes in appropriately selected patients, at the expense of a roughly 10%–13% re-operation rate and 27% overall complication rate [20]. Importantly, timing in chronic non-union is less critical than in acute flail chest; most patients in published series underwent surgery 12–24 months after injury [17]. Our first patient was treated at 6 months once it was clear that pain, mechanical symptoms, and functional limitation persisted, whereas the second patient proceeded to surgery after several months of refractory symptoms and clear imaging evidence of cartilage pseudarthrosis.

### Technical considerations

#### Posterior rib non-union and costal margin rupture

Posterior rib non-unions are technically challenging because of their deep location, proximity to the spine, and substantial muscle coverage. Several groups have emphasized the value of preoperative thin-slice CT with three-dimensional reconstruction, intraoperative fluoroscopy, and—more recently—3D-printed models to localize non-unions and plan incisions [1, 2, 24, 25]. A muscle-sparing lateral or parascapular approach allows exposure while preserving scapulothoracic mechanics. Debridement of fibrous tissue and sclerotic bone to healthy, bleeding cortices is essential, and many authors advocate opening the medullary canal to enhance endosteal blood flow [17, 24]. Rigid fixation is then achieved with low-profile locking plates or, in selected cases, intramedullary splints. Autologous bone graft is recommended when a gap remains after debridement [15, 17, 24, 25].

Our first case illustrates an additional layer of complexity: posterior pseudarthrosis can alter chest wall mechanics sufficiently to precipitate secondary costal margin rupture with intercostal hernia. The Sheffield classification has formalized these lesions, distinguishing patterns of costal margin rupture with or without intercostal hernia (costal margin rupture with intercostal hernia (CMR-IH), trans diaphragmatic intercostal hernia (TDIH)) and their association with posterior, often cough-induced rib fractures [4, 5, 13, 20]. Surgical management has evolved from simple suture repair to more robust reconstructions using titanium buttress plates on the costal arch, intercostal nerve-sparing suture techniques, and double-layer mesh reinforcement, often combined with rib plating or SSNURF when posterior fractures are present [12, 13]. Our technique—posterior plating with local grafting, costal arch buttress plates, fibre-tape repair of the intercostal defect routed through plate holes, and double-layer mesh—aligns with these contemporary concepts and is intended to restore both structural integrity and physiological load transfer across the costal margin.

#### Parasternal cartilage pseudarthrosis and sternal region

Parasternal costal cartilage injuries are uncommon and often radiographically occult. MRI and dynamic ultrasound are particularly useful to detect cartilage rupture, peri-cartilaginous oedema, and non-union at the costosternal junction [8–10, 25]. When symptoms are severe and persistent, options range from excision of the pseudarthrosis with soft-tissue repair to rigid osteosynthesis bridging the affected cartilage to the sternum or adjacent ribs.

Analogous to sternal non-union, where parallel locking plates have largely supplanted wire cerclage because of superior stability and lower non-union rates [18, 26, 27], we used a low-profile locking plate to bridge the third rib, costal cartilage, and sternum. Contouring to the chest wall, unicortical screw placement in the sternum and rib, and careful handling of perichondrium are important to minimize the risk of hardware prominence or cartilage fracture. The use of MRI-compatible titanium implants is particularly attractive in younger patients or those with prior cardiac surgery, as it preserves access to high-quality postoperative cross-sectional imaging [14]. Local cancellous grafting at the bone–cartilage interface, as in our case, is extrapolated from sternal non-union and rib non-union literature and may further enhance healing [17, 23, 24, 26].

#### Role of intramedullary splints and minimally invasive techniques

Although we did not employ intramedullary devices in these two cases, there is growing interest in splint-based and minimally invasive approaches for selected non-unions, particularly in posterior ribs. Case series have shown that intramedullary rib splints can be inserted through small incisions, with satisfactory pain relief and union in both acute and chronic settings [21, 25, 28]. Ultrasound guidance may aid in precise localization of fracture sites and reduce incision length [16, 18, 19]. Current evidence suggests that these techniques are best reserved for simple, single-level non-unions without major deformity; more complex patterns like those we report still require open, plate-based reconstruction.

#### Outcomes and how our cases add to current evidence

Across published case series, surgical stabilization of symptomatic rib non-union achieves radiographic union in >90% of patients, with substantial reductions in pain scores and improved return-to-work rates [1, 2, 7, 11, 15, 17, 24]. Implant failure occurs in roughly 10%–12%, infection in 4%–5%, and re-operation in about 13% of cases [17]. Long-term follow-up suggests that most patients remain satisfied despite residual mild pain or reduced pulmonary function in some [15]. For costal margin rupture with hernia, evolution from simple suture closure to plate-and-mesh reconstructions has markedly reduced hernia recurrence and improved quality of life [12, 13]. For sternal and parasternal non-union, small series and case reports show that rigid plating provides rapid pain relief, improved cosmesis, and low re-operation rates when combined with adequate debridement and, when necessary, soft-tissue reconstruction [14, 18, 22, 23, 26, 27].

Our report adds several points to this growing body of literature:

- **Posterior rib non-union with secondary costal margin rupture and hernia**: while posterior rib non-unions and costal margin injuries have each been described, detailed operative management of their combined occurrence is rarely reported. Our case demonstrates that simultaneous posterior plating with autograft and advanced costal margin reconstruction can be safely performed in a single stage, with excellent pain relief, restoration of pulmonary function, and no recurrence of intercostal hernia at mid-term follow-up.
- **MRI-compatible trans-costosternal plating for isolated parasternal cartilage pseudarthrosis**: isolated third-rib parasternal cartilage non-union in a young adult, with normal radiography and initial CT, is an unusual presentation. We show that careful multimodal imaging with repeat focused MRI and dynamic ultrasound can establish the diagnosis, and that low-profile locking-plate osteosynthesis bridging rib, cartilage, and sternum is feasible, provides immediate mechanical stability, and allows early return to full activity without compromising future imaging.
- **Diagnostic pathway emphasizing ultrasound**: both cases underscore the complementary role of dynamic ultrasound alongside CT and MRI. In keeping with recent radiological and surgical reviews, our experience supports the notion that ultrasound is superior to conventional radiography and often more informative than static CT for detecting costal cartilage and costal margin instability, particularly in patients with persistent symptoms and inconclusive initial imaging [3, 4, 8, 10, 16, 29].

## Conclusion

In conclusion, complex non-union of the posterior ribs, parasternal costal cartilage, and costal margin can cause substantial morbidity but is amenable to definitive surgical treatment. Modern locking plates, intramedullary devices, and mesh techniques, applied within a multidisciplinary framework, allow restoration of chest wall stability, marked pain reduction, and early functional recovery in carefully selected patients. Prospective multicentre studies are needed to refine indications, compare different fixation strategies, and define optimal timing and imaging algorithms for these challenging but increasingly recognized injuries.

## Data Availability

All data generated or analyzed during this study are included in this published article and its supplemental information files.

## References

[1] van Wijck SFM, van Lieshout EMM, Prins JTH et al. Outcome after surgical stabilization of symptomatic rib fracture nonunion: a multicenter retrospective case series. Eur J Trauma Emerg Surg 2022;48:2783–93. 10.1007/s00068-021-01867-x 35088110 PMC9360056

[2] de Jong MB, Houwert RM, van Heerde S et al. Surgical treatment of rib fracture nonunion: a single center experience. Injury 2018;49:599–603. 10.1016/j.injury.2018.01.004 29402425

[3] Nummela MT, Pyhältö TT, Bensch FV et al. Costal cartilage fractures in blunt polytrauma patients—a prospective clinical and radiological follow-up study. Emerg Radiol 2022;29:845–54. 10.1007/s10140-022-02066-w 35661281 PMC9458556

[4] Gooseman MR, Rawashdeh M, Mattam K et al. Unifying classification for transdiaphragmatic intercostal hernia and other costal margin injuries. Eur J Cardiothorac Surg 2019;56:150–8. 10.1093/ejcts/ezz020 30770701

[5] Mattam K, Wijerathne P, Rao JN et al. Radiological insights into costal margin rupture injuries: patterns of associated rib and costal cartilage fractures. Eur J Trauma Emerg Surg 2025;51:112. 10.1007/s00068-025-02781-2 39966168

[6] DeGenova DT, Miller KB, McClure TT et al. Operative fixation of rib fracture nonunions. Arch Orthop Trauma Surg 2023;143:3047–54. 10.1007/s00402-022-04540-z 35829736

[7] Buehler KE, Wilshire CL, Bograd AJ et al. Rib plating offers favorable outcomes in patients with chronic nonunion of prior rib fractures. Ann Thorac Surg 2020;110:993–7. 10.1016/j.athoracsur.2020.03.075 32353437

[8] Malghem J, Vande Berg B, Lecouvet F et al. Costal cartilage fractures as revealed on CT and sonography. AJR Am J Roentgenol 2001;176:429–32. 10.2214/ajr.176.2.1760429 11159088

[9] Ogunleye TD, Carlson DA, Thomas CN et al. Outcomes after operative reconstruction of symptomatic rib nonunions. J Orthop Trauma 2022;36:e161–6. 10.1097/BOT.0000000000002275 35594515

[10] Subhas N, Kline MJ, Moskal MJ et al. MRI evaluation of costal cartilage injuries. AJR Am J Roentgenol 2008;191:129–32. 10.2214/AJR.07.3396 18562735

[11] Fabricant L, Ham B, Mullins R et al. Prospective clinical trial of surgical intervention for painful rib fracture nonunion. Am Surg 2014;80:580–6. 10.1177/000313481408000622 24887796

[12] Wijerathne P, Rao JN, Wijffels MME et al. Surgical management of costal margin rupture associated with intercostal hernia: evolution of techniques. J Trauma Acute Care Surg 2024;97:861–8. 10.1097/TA.0000000000004440 39733286

[13] Byers JL, Rao JN, Socci L et al. Costal margin injuries and trans-diaphragmatic intercostal hernia: presentation, management and outcomes according to the Sheffield classification. J Trauma Acute Care Surg 2023;95:839–45. 10.1097/TA.0000000000004068 37533145

[14] Intihar U, Zeleznik J, Brajlih T et al. Sternal reconstruction using 3D-printed titanium custom-made prosthesis for sternal dehiscence after cardiac surgery. Heart Surg Forum 2023;26:E160–3. 10.1532/hsf.5151 36972602

[15] Nilsson J, Caragounis EC. Long-term outcome after surgical management of symptomatic non-union rib fractures. Injury 2024;55:111297. 10.1016/j.injury.2023.111297 38151437

[16] Martin TJ, Cao J, Benoit E et al. Optimizing surgical stabilization of rib fractures using intraoperative ultrasound localization. J Trauma Acute Care Surg 2021;91:369–74. 10.1097/TA.0000000000003262 33938512

[17] Adams-McGavin RC, Naveed A, Kishibe T et al. Management of non-union of rib fractures secondary to trauma: a scoping review. Injury 2024;55:111553. 10.1016/j.injury.2024.111553 38762403

[18] Ceresa F, Casablanca G, Patanè F. Complicated sternal dehiscence treated with the Strasbourg Thoracic Osteosyntheses System (STRATOS) and transposition of greater omentum: a case report. J Cardiothorac Surg 2010;5:53. 10.1186/1749-8090-5-53 20584311 PMC2903575

[19] Vossler JD, Zhao FZ. Intercostal nerve cryoablation for control of traumatic rib fracture pain: a case report. Trauma Case Rep 2019;23:100229. 10.1016/j.tcr.2019.100229 31388539 PMC6676040

[20] Forrester JD, Bauman ZM, Cole PA et al. Chest Wall Injury Society (CWIS) recommendation for surgical stabilization of non-united rib fractures (SSNURF) to decrease pain, reduce opiate use, and improve patient reported outcomes in patients with rib fracture nonunion after trauma. J Trauma Acute Care Surg 2023;95:943–50. 10.1097/TA.0000000000004083 37728432

[21] Raveglia F, Libretti L, Cioffi U et al. Minimally invasive surgical management of chronic cough-induced rib fracture non-union: a case report. Am J Case Rep 2024;25:e943222. 10.12659/AJCR.943222 38917052 PMC11334099

[22] Kehoe JD, Barrett S, Higgins P et al. Sternal plating for traumatic sternal non-union: a case series and literature review. Ir Med J 2024;117:1007. 39377373

[23] Spering C, von Hammerstein-Equord A, Lehmann W et al. Osteosyntheseverfahren bei Thoraxwandinstabilität. Oper Orthop Traumatol 2021;33:262–84. 10.1007/s00064-020-00688-2 33289872 PMC7722258

[24] Hernandez MC, Reisenauer JS, Aho JM et al. Bone autograft coupled with locking plates repairs symptomatic rib fracture nonunions. Am Surg 2018;84:844–50. 10.1177/000313481808400630 29981613

[25] Bergquist JR, Morris JM, Matsumoto JM et al. 3D printed modeling contributes to reconstruction of complex chest wall instability. Trauma Case Rep 2019;22:100218. 10.1016/j.tcr.2019.100218 31249855 PMC6584793

[26] Salehi F, Niusha S, Saghebi SR et al. Technical details of surgical treatment of a severely displaced sternal fracture. Tanaffos 2019;18:365–8. 32607119 PMC7309882

[27] Voss B, Bauernschmitt R, Will A et al. Sternal reconstruction with titanium plates in complicated sternal dehiscence: mid-term results. Eur J Cardiothorac Surg 2008;34:139–45. 10.1016/j.ejcts.2008.03.030 18455410

[28] Leasia K, Prins JTH, Lawless R et al. Door to door in 24: factors that allow surgical stabilization of rib fractures within 24 hours of admission. J Thorac Dis 2023;15:5922–30. 10.21037/jtd-23-857 38090313 PMC10713285

[29] Mattox R, Reckelhoff KE, Welk AB et al. Sonography of occult rib and costal cartilage fractures: a case series. J Chiropr Med 2014;13:139–43. 10.1016/j.jcm.2014.06.008 25685124 PMC4322009

